# Search for long-lived heavy charged particles using a ring imaging Cherenkov technique at LHCb

**DOI:** 10.1140/epjc/s10052-015-3809-7

**Published:** 2015-12-15

**Authors:** R. Aaij, B. Adeva, M. Adinolfi, A. Affolder, Z. Ajaltouni, S. Akar, J. Albrecht, F. Alessio, M. Alexander, S. Ali, G. Alkhazov, P. Alvarez Cartelle, A. A. Alves Jr, S. Amato, S. Amerio, Y. Amhis, L. An, L. Anderlini, J. Anderson, M. Andreotti, J. E. Andrews, R. B. Appleby, O. Aquines Gutierrez, F. Archilli, P. d’Argent, A. Artamonov, M. Artuso, E. Aslanides, G. Auriemma, M. Baalouch, S. Bachmann, J. J. Back, A. Badalov, C. Baesso, W. Baldini, R. J. Barlow, C. Barschel, S. Barsuk, W. Barter, V. Batozskaya, V. Battista, A. Bay, L. Beaucourt, J. Beddow, F. Bedeschi, I. Bediaga, L. J. Bel, I. Belyaev, E. Ben-Haim, G. Bencivenni, S. Benson, J. Benton, A. Berezhnoy, R. Bernet, A. Bertolin, M.-O. Bettler, M. van Beuzekom, A. Bien, S. Bifani, T. Bird, A. Birnkraut, A. Bizzeti, T. Blake, F. Blanc, J. Blouw, S. Blusk, V. Bocci, A. Bondar, N. Bondar, W. Bonivento, S. Borghi, A. Borgia, M. Borsato, T. J. V. Bowcock, E. Bowen, C. Bozzi, D. Brett, M. Britsch, T. Britton, J. Brodzicka, N. H. Brook, A. Bursche, J. Buytaert, S. Cadeddu, R. Calabrese, M. Calvi, M. Calvo Gomez, P. Campana, D. Campora Perez, L. Capriotti, A. Carbone, G. Carboni, R. Cardinale, A. Cardini, P. Carniti, L. Carson, K. Carvalho Akiba, R. Casanova Mohr, G. Casse, L. Cassina, L. Castillo Garcia, M. Cattaneo, Ch. Cauet, G. Cavallero, R. Cenci, M. Charles, Ph. Charpentier, M. Chefdeville, S. Chen, S. F. Cheung, N. Chiapolini, M. Chrzaszcz, X. Cid Vidal, G. Ciezarek, P. E. L. Clarke, M. Clemencic, H. V. Cliff, J. Closier, V. Coco, J. Cogan, E. Cogneras, V. Cogoni, L. Cojocariu, G. Collazuol, P. Collins, A. Comerma-Montells, A. Contu, A. Cook, M. Coombes, S. Coquereau, G. Corti, M. Corvo, I. Counts, B. Couturier, G. A. Cowan, D. C. Craik, A. Crocombe, M. Cruz Torres, S. Cunliffe, R. Currie, C. D’Ambrosio, J. Dalseno, P. N. Y. David, A. Davis, K. De Bruyn, S. De Capua, M. De Cian, J. M. De Miranda, L. De Paula, W. De Silva, P. De Simone, C. T. Dean, D. Decamp, M. Deckenhoff, L. Del Buono, N. Déléage, D. Derkach, O. Deschamps, F. Dettori, B. Dey, A. Di Canto, F. Di Ruscio, H. Dijkstra, S. Donleavy, F. Dordei, M. Dorigo, A. Dosil Suárez, D. Dossett, A. Dovbnya, K. Dreimanis, G. Dujany, F. Dupertuis, P. Durante, R. Dzhelyadin, A. Dziurda, A. Dzyuba, S. Easo, U. Egede, V. Egorychev, S. Eidelman, S. Eisenhardt, U. Eitschberger, R. Ekelhof, L. Eklund, I. El Rifai, Ch. Elsasser, S. Ely, S. Esen, H. M. Evans, T. Evans, A. Falabella, C. Färber, C. Farinelli, N. Farley, S. Farry, R. Fay, D. Ferguson, V. Fernandez Albor, F. Ferrari, F. Ferreira Rodrigues, M. Ferro-Luzzi, S. Filippov, M. Fiore, M. Fiorini, M. Firlej, C. Fitzpatrick, T. Fiutowski, P. Fol, M. Fontana, F. Fontanelli, R. Forty, O. Francisco, M. Frank, C. Frei, M. Frosini, J. Fu, E. Furfaro, A. Gallas Torreira, D. Galli, S. Gallorini, S. Gambetta, M. Gandelman, P. Gandini, Y. Gao, J. García Pardiñas, J. Garofoli, J. Garra Tico, L. Garrido, D. Gascon, C. Gaspar, R. Gauld, L. Gavardi, G. Gazzoni, A. Geraci, D. Gerick, E. Gersabeck, M. Gersabeck, T. Gershon, Ph. Ghez, A. Gianelle, S. Gianì, V. Gibson, L. Giubega, V. V. Gligorov, C. Göbel, D. Golubkov, A. Golutvin, A. Gomes, C. Gotti, M. Grabalosa Gándara, R. Graciani Diaz, L. A. Granado Cardoso, E. Graugés, E. Graverini, G. Graziani, A. Grecu, E. Greening, S. Gregson, P. Griffith, L. Grillo, O. Grünberg, B. Gui, E. Gushchin, Yu. Guz, T. Gys, C. Hadjivasiliou, G. Haefeli, C. Haen, S. C. Haines, S. Hall, B. Hamilton, T. Hampson, X. Han, S. Hansmann-Menzemer, N. Harnew, S. T. Harnew, J. Harrison, J. He, T. Head, V. Heijne, K. Hennessy, P. Henrard, L. Henry, J. A. Hernando Morata, E. van Herwijnen, M. Heß, A. Hicheur, D. Hill, M. Hoballah, C. Hombach, W. Hulsbergen, T. Humair, N. Hussain, D. Hutchcroft, D. Hynds, M. Idzik, P. Ilten, R. Jacobsson, A. Jaeger, J. Jalocha, E. Jans, A. Jawahery, F. Jing, M. John, D. Johnson, C. R. Jones, C. Joram, B. Jost, N. Jurik, S. Kandybei, W. Kanso, M. Karacson, T. M. Karbach, S. Karodia, M. Kelsey, I. R. Kenyon, M. Kenzie, T. Ketel, B. Khanji, C. Khurewathanakul, S. Klaver, K. Klimaszewski, O. Kochebina, M. Kolpin, I. Komarov, R. F. Koopman, P. Koppenburg, L. Kravchuk, K. Kreplin, M. Kreps, G. Krocker, P. Krokovny, F. Kruse, W. Kucewicz, M. Kucharczyk, V. Kudryavtsev, K. Kurek, T. Kvaratskheliya, V. N. La Thi, D. Lacarrere, G. Lafferty, A. Lai, D. Lambert, R. W. Lambert, G. Lanfranchi, C. Langenbruch, B. Langhans, T. Latham, C. Lazzeroni, R. Le Gac, J. van Leerdam, J. P. Lees, R. Lefèvre, A. Leflat, J. Lefrançois, O. Leroy, T. Lesiak, B. Leverington, Y. Li, T. Likhomanenko, M. Liles, R. Lindner, C. Linn, F. Lionetto, B. Liu, S. Lohn, I. Longstaff, J. H. Lopes, D. Lucchesi, H. Luo, A. Lupato, E. Luppi, O. Lupton, F. Machefert, I. V. Machikhiliyan, F. Maciuc, O. Maev, S. Malde, A. Malinin, G. Manca, G. Mancinelli, P. Manning, A. Mapelli, J. Maratas, J. F. Marchand, U. Marconi, C. Marin Benito, P. Marino, R. Märki, J. Marks, G. Martellotti, M. Martinelli, D. Martinez Santos, F. Martinez Vidal, D. Martins Tostes, A. Massafferri, R. Matev, Z. Mathe, C. Matteuzzi, A. Mauri, B. Maurin, A. Mazurov, M. McCann, J. McCarthy, A. McNab, R. McNulty, B. McSkelly, B. Meadows, F. Meier, M. Meissner, M. Merk, D. A. Milanes, M. N. Minard, D. S. Mitzel, J. Molina Rodriguez, S. Monteil, M. Morandin, P. Morawski, A. Mordà, M. J. Morello, J. Moron, A. B. Morris, R. Mountain, F. Muheim, J. Müller, K. Müller, V. Müller, M. Mussini, B. Muster, P. Naik, T. Nakada, R. Nandakumar, I. Nasteva, M. Needham, N. Neri, S. Neubert, N. Neufeld, M. Neuner, A. D. Nguyen, T. D. Nguyen, C. Nguyen-Mau, V. Niess, R. Niet, N. Nikitin, T. Nikodem, A. Novoselov, D. P. O’Hanlon, A. Oblakowska-Mucha, V. Obraztsov, S. Ogilvy, O. Okhrimenko, R. Oldeman, C. J. G. Onderwater, B. Osorio Rodrigues, J. M. Otalora Goicochea, A. Otto, P. Owen, A. Oyanguren, A. Palano, F. Palombo, M. Palutan, J. Panman, A. Papanestis, M. Pappagallo, L. L. Pappalardo, C. Parkes, G. Passaleva, G. D. Patel, M. Patel, C. Patrignani, A. Pearce, A. Pellegrino, G. Penso, M. Pepe Altarelli, S. Perazzini, P. Perret, L. Pescatore, K. Petridis, A. Petrolini, E. Picatoste Olloqui, B. Pietrzyk, T. Pilař, D. Pinci, A. Pistone, S. Playfer, M. Plo Casasus, T. Poikela, F. Polci, A. Poluektov, I. Polyakov, E. Polycarpo, A. Popov, D. Popov, B. Popovici, C. Potterat, E. Price, J. D. Price, J. Prisciandaro, A. Pritchard, C. Prouve, V. Pugatch, A. Puig Navarro, G. Punzi, W. Qian, R. Quagliani, B. Rachwal, J. H. Rademacker, B. Rakotomiaramanana, M. Rama, M. S. Rangel, I. Raniuk, N. Rauschmayr, G. Raven, F. Redi, S. Reichert, M. M. Reid, A. C. dos Reis, S. Ricciardi, S. Richards, M. Rihl, K. Rinnert, V. Rives Molina, P. Robbe, A. B. Rodrigues, E. Rodrigues, P. Rodriguez Perez, S. Roiser, V. Romanovsky, A. Romero Vidal, M. Rotondo, J. Rouvinet, T. Ruf, H. Ruiz, P. Ruiz Valls, J. J. Saborido Silva, N. Sagidova, P. Sail, B. Saitta, V. Salustino Guimaraes, C. Sanchez Mayordomo, B. Sanmartin Sedes, R. Santacesaria, C. Santamarina Rios, E. Santovetti, A. Sarti, C. Satriano, A. Satta, D. M. Saunders, D. Savrina, M. Schiller, H. Schindler, M. Schlupp, M. Schmelling, T. Schmelzer, B. Schmidt, O. Schneider, A. Schopper, M. H. Schune, R. Schwemmer, B. Sciascia, A. Sciubba, A. Semennikov, I. Sepp, N. Serra, J. Serrano, L. Sestini, P. Seyfert, M. Shapkin, I. Shapoval, Y. Shcheglov, T. Shears, L. Shekhtman, V. Shevchenko, A. Shires, R. Silva Coutinho, G. Simi, M. Sirendi, N. Skidmore, I. Skillicorn, T. Skwarnicki, E. Smith, E. Smith, J. Smith, M. Smith, H. Snoek, M. D. Sokoloff, F. J. P. Soler, F. Soomro, D. Souza, B. Souza De Paula, B. Spaan, P. Spradlin, S. Sridharan, F. Stagni, M. Stahl, S. Stahl, O. Steinkamp, O. Stenyakin, F. Sterpka, S. Stevenson, S. Stoica, S. Stone, B. Storaci, S. Stracka, M. Straticiuc, U. Straumann, R. Stroili, L. Sun, W. Sutcliffe, K. Swientek, S. Swientek, V. Syropoulos, M. Szczekowski, P. Szczypka, T. Szumlak, S. T’Jampens, T. Tekampe, M. Teklishyn, G. Tellarini, F. Teubert, C. Thomas, E. Thomas, J. van Tilburg, V. Tisserand, M. Tobin, J. Todd, S. Tolk, L. Tomassetti, D. Tonelli, S. Topp-Joergensen, N. Torr, E. Tournefier, S. Tourneur, K. Trabelsi, M. T. Tran, M. Tresch, A. Trisovic, A. Tsaregorodtsev, P. Tsopelas, N. Tuning, M. Ubeda Garcia, A. Ukleja, A. Ustyuzhanin, U. Uwer, C. Vacca, V. Vagnoni, G. Valenti, A. Vallier, R. Vazquez Gomez, P. Vazquez Regueiro, C. Vázquez Sierra, S. Vecchi, J. J. Velthuis, M. Veltri, G. Veneziano, M. Vesterinen, B. Viaud, D. Vieira, M. Vieites Diaz, X. Vilasis-Cardona, A. Vollhardt, D. Volyanskyy, D. Voong, A. Vorobyev, V. Vorobyev, C. Voß, J. A. de Vries, R. Waldi, C. Wallace, R. Wallace, J. Walsh, S. Wandernoth, J. Wang, D. R. Ward, N. K. Watson, D. Websdale, A. Weiden, M. Whitehead, D. Wiedner, G. Wilkinson, M. Wilkinson, M. Williams, M. P. Williams, M. Williams, F. F. Wilson, J. Wimberley, J. Wishahi, W. Wislicki, M. Witek, G. Wormser, S. A. Wotton, S. Wright, K. Wyllie, Y. Xie, Z. Xu, Z. Yang, X. Yuan, O. Yushchenko, M. Zangoli, M. Zavertyaev, L. Zhang, Y. Zhang, A. Zhelezov, A. Zhokhov, L. Zhong

**Affiliations:** Centro Brasileiro de Pesquisas Físicas (CBPF), Rio de Janeiro, Brazil; Universidade Federal do Rio de Janeiro (UFRJ), Rio de Janeiro, Brazil; Center for High Energy Physics, Tsinghua University, Beijing, China; LAPP, Université Savoie Mont-Blanc, CNRS/IN2P3, Annecy-Le-Vieux, France; Clermont Université, Université Blaise Pascal, CNRS/IN2P3, LPC, Clermont-Ferrand, France; CPPM, Aix-Marseille Université, CNRS/IN2P3, Marseille, France; LAL, Université Paris-Sud, CNRS/IN2P3, Orsay, France; LPNHE, Université Pierre et Marie Curie, Université Paris Diderot, CNRS/IN2P3, Paris, France; Fakultät Physik, Technische Universität Dortmund, Dortmund, Germany; Max-Planck-Institut für Kernphysik (MPIK), Heidelberg, Germany; Physikalisches Institut, Ruprecht-Karls-Universität Heidelberg, Heidelberg, Germany; School of Physics, University College Dublin, Dublin, Ireland; Sezione INFN di Bari, Bari, Italy; Sezione INFN di Bologna, Bologna, Italy; Sezione INFN di Cagliari, Cagliari, Italy; Sezione INFN di Ferrara, Ferrara, Italy; Sezione INFN di Firenze, Florence, Italy; Laboratori Nazionali dell’INFN di Frascati, Frascati, Italy; Sezione INFN di Genova, Genoa, Italy; Sezione INFN di Milano Bicocca, Milan, Italy; Sezione INFN di Milano, Milan, Italy; Sezione INFN di Padova, Padua, Italy; Sezione INFN di Pisa, Pisa, Italy; Sezione INFN di Roma Tor Vergata, Rome, Italy; Sezione INFN di Roma La Sapienza, Rome, Italy; Henryk Niewodniczanski Institute of Nuclear Physics Polish Academy of Sciences, Kraków, Poland; Faculty of Physics and Applied Computer Science, AGH, University of Science and Technology, Kraków, Poland; National Center for Nuclear Research (NCBJ), Warsaw, Poland; Horia Hulubei National Institute of Physics and Nuclear Engineering, Bucharest-Magurele, Romania; Petersburg Nuclear Physics Institute (PNPI), Gatchina, Russia; Institute of Theoretical and Experimental Physics (ITEP), Moscow, Russia; Institute of Nuclear Physics, Moscow State University (SINP MSU), Moscow, Russia; Institute for Nuclear Research of the Russian Academy of Sciences (INR RAN), Moscow, Russia; Budker Institute of Nuclear Physics (SB RAS), Novosibirsk State University, Novosibirsk, Russia; Institute for High Energy Physics (IHEP), Protvino, Russia; Universitat de Barcelona, Barcelona, Spain; Universidad de Santiago de Compostela, Santiago de Compostela, Spain; European Organization for Nuclear Research (CERN), Geneva, Switzerland; Ecole Polytechnique Fédérale de Lausanne (EPFL), Lausanne, Switzerland; Physik-Institut, Universität Zürich, Zurich, Switzerland; Nikhef National Institute for Subatomic Physics, Amsterdam, The Netherlands; Nikhef National Institute for Subatomic Physics, VU University Amsterdam, Amsterdam, The Netherlands; NSC Kharkiv Institute of Physics and Technology (NSC KIPT), Kharkiv, Ukraine; Institute for Nuclear Research of the National Academy of Sciences (KINR), Kyiv, Ukraine; University of Birmingham, Birmingham, UK; H.H. Wills Physics Laboratory, University of Bristol, Bristol, UK; Cavendish Laboratory, University of Cambridge, Cambridge, UK; Department of Physics, University of Warwick, Coventry, UK; STFC Rutherford Appleton Laboratory, Didcot, UK; School of Physics and Astronomy, University of Edinburgh, Edinburgh, UK; School of Physics and Astronomy, University of Glasgow, Glasgow, UK; Oliver Lodge Laboratory, University of Liverpool, Liverpool, UK; Imperial College London, London, UK; School of Physics and Astronomy, University of Manchester, Manchester, UK; Department of Physics, University of Oxford, Oxford, UK; Massachusetts Institute of Technology, Cambridge, MA USA; University of Cincinnati, Cincinnati, OH USA; University of Maryland, College Park, MD USA; Syracuse University, Syracuse, NY USA; Pontifícia Universidade Católica do Rio de Janeiro (PUC-Rio), Rio de Janeiro, Brazil; Institute of Particle Physics, Central China Normal University, Wuhan, Hubei China; Departamento de Fisica, Universidad Nacional de Colombia, Bogota, Colombia; Institut für Physik, Universität Rostock, Rostock, Germany; National Research Centre Kurchatov Institute, Moscow, Russia; Yandex School of Data Analysis, Moscow, Russia; Instituto de Fisica Corpuscular (IFIC), Universitat de Valencia-CSIC, Valencia, Spain; Van Swinderen Institute, University of Groningen, Groningen, The Netherlands

## Abstract

A search is performed for heavy long-lived charged particles using 3.0 $${\rm fb}^{-1}$$ of proton–proton collisions collected at $$\sqrt{s}$$$$=$$ 7 and 8  TeV with the LHCb detector. The search is mainly based on the response of the ring imaging Cherenkov detectors to distinguish the heavy, slow-moving particles from muons. No evidence is found for the production of such long-lived states. The results are expressed as limits on the Drell–Yan production of pairs of long-lived particles, with both particles in the LHCb pseudorapidity acceptance, $$1.8 < \eta < 4.9$$. The mass-dependent cross-section upper limits are in the range 2–4 fb (at 95 % CL) for masses between 14 and 309 $${\mathrm {\,GeV\!/}c^2}$$.

## Introduction

Several extensions of the Standard Model (SM) propose the existence of charged massive stable particles (CMSP). Stable particles, in this context, are long-lived particles that can travel through a detector without decaying. These particles can have long lifetimes for a variety of reasons, e.g. a new (approximately) conserved quantum number, a weak coupling or a limited phase space in any allowed decay. In supersymmetric (SUSY) models, CMSPs can be sleptons ($\widetilde{\ell }$), charginos, or R-hadrons. R-hadrons are colourless states combining squarks ($\widetilde{q}$) or gluinos ($\widetilde{g}$) and SM quarks or gluons. In the gauge-mediated supersymmetry breaking (GMSB) model [1–3] the breakdown of SUSY is mediated by gauge interactions and can occur at a relatively low energy scale. For a particular range of parameter space in the minimal model (mGMSB) the next-to-lightest supersymmetric particle can be a long-lived stau ($\widetilde{\tau _1}$), with a mass of the order of 100${\mathrm {\,GeV\!/}c^2}$ or higher. The $\widetilde{\tau _1}$ is the lightest mass eigenstate, resulting from the mixture of right-handed and left-handed superpartners of the $\tau$, dominated by the right-handed component.

A CMSP loses energy mainly via ionisation; strongly interacting CMSPs are not considered here. In a detector such as LHCb a CMSP with a kinetic energy above about 5  GeV should be able to traverse the muon chambers. Those particles would often be produced with a relatively low velocity and could be identified by their time-of-flight, and by their specific energy loss, $\mathrm {d}E/\mathrm {d}x$, in the detectors; Cherenkov radiation would be absent in Cherenkov counters tuned for ultra-relativistic particles.

Several experiments have searched for CMSPs [4–12]. With the exception of DELPHI [5], which had Cherenkov counters, the analyses are based on $\mathrm {d}E/\mathrm {d}x$ and time-of-flight measurements. The primary interest here is to show the potential of the identification technique based on ring imaging Cherenkov (RICH) detectors, in addition to the exploration of the forward pseudorapidity region only partially covered by the central detectors at the Tevatron and the LHC.

The analysis described in this study is mainly based on the absence of Cherenkov radiation in the RICH detectors. This technique is used to search for pairs of CMSPs in LHCb, produced by a Drell–Yan mechanism.

## The LHCb detector and the detection of slow particles

The LHCb detector [13, 14] is a single-arm forward spectrometer covering the approximate pseudorapidity range $$1.8<\eta <4.9$$, designed for the study of particles containing $$\mathrm {b} $$ or $$\mathrm {c} $$ quarks. The detector includes a high-precision tracking system consisting of a silicon-strip vertex detector (the vertex locator, VELO) surrounding the proton–proton interaction region [15], a large-area silicon-strip detector located upstream of a dipole magnet with a bending power of about $$4\mathrm{\,Tm}$$, and three stations of silicon-strip detectors and straw drift tubes [16] placed downstream of the magnet. The tracking system provides a measurement of momentum, *p* , of charged particles with a relative uncertainty that varies from 0.5 % at low momentum to 1.0 % at 200 $${\mathrm {\,GeV\!/}c}$$. The minimum distance of a track to a primary vertex (PV), the impact parameter (IP), is measured with a resolution of $$(15+29/$$$$p_\mathrm{T}$$ )$${\,\upmu {\mathrm{m}}}$$, where $$p_\mathrm{T}$$ is the component of the momentum transverse to the beam, in $${\mathrm {\,GeV\!/}c}$$. Photons, electrons and hadrons are identified by a calorimeter system consisting of scintillating-pad and preshower detectors, an electromagnetic calorimeter and a hadronic calorimeter. Muons are identified by a system composed of alternating layers of iron and multiwire proportional chambers [17].

Different types of charged particles are distinguished using information from two RICH detectors [18]. The RICH system, which plays a crucial role in this analysis, consists of an upstream detector with silica aerogel and $\mathrm C_4F_{10}$ gas radiators, positioned directly after the VELO, and a downstream detector with a $\mathrm CF_4$ gas radiator, located just after the tracking system.

The online event selection is performed by a trigger [19], which consists of a hardware stage, based on information from the calorimeter and muon systems, followed by a software stage, which applies a full event reconstruction.

The analysis presented here is based on two data sets collected in 2011 and 2012 corresponding to integrated luminosities of 1.0  and 2.0 $bad hbox^{-1}$ from proton–proton collisions recorded at centre-of-mass energies of 7 and 8  TeV, respectively.

**Fig. 1:**
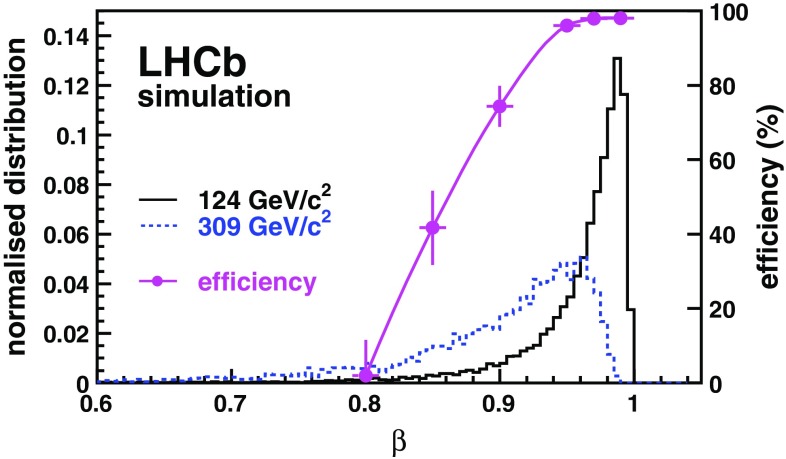
CMSP velocity spectrum for the CMSP masses of 124 and 309 ${\mathrm {\,GeV\!/}c^2}$. The proton–proton centre-of-mass energy is 7 TeV. The *dots with error bars* show the efficiency to detect tracks as a function of the $\beta$ of the particle (*right scale*)

In the production process considered, CMSPs can have velocities $\beta \equiv v/c$ as low as 0.7, and their arrival time at the subdetectors can differ by several nanoseconds with respect to lighter particles with $\beta \simeq 1$. For illustration, the $\beta$ spectrum is shown in Fig. 1, for two values of the CMSP mass, at centre-of-mass energy of 7  TeV. The effects of such delayed detection on the efficiencies of the subdetectors are determined from simulation in which the timing information is modelled according to dedicated electronic measurements and tests in beam. The muon chambers have the largest inefficiency for slow-particle reconstruction. The maximal delay for a particle to be accepted by the front-end electronics is 12.5 ns [17]. In the most downstream muon chamber, this delay corresponds to the arrival of a particle with $\beta =0.83$. To be identified as a muon, the charged particle must be associated with hits in the last four muon chambers, a requirement that particles with $\beta < 0.8$ fail to meet. The large time-of-flight can also bias the reconstructed position of the particle passing through the tracker straw tubes, which accept a maximal drift-time of about 35 ns for tracks passing close to the straw radius of 2.5 mm [16]. These combined effects result in a vanishingly small reconstruction efficiency for particles with $\beta < 0.8$ but an efficiency above 95 % if $\beta > 0.95$, as shown in Fig. 1.

## Simulation

### CMSP signal

The adopted framework is stau pair production, $\widetilde{\tau _1} ^{+} \widetilde{\tau _1} ^{-}$, in mGMSB via a Drell–Yan process. Pairs of CMSPs originating from cascade decays of heavier particles are explicitly not considered. In the following the symbol $\widetilde{\tau _1}$ is used when the context is explicitly the mGMSB model, while CMSP is kept for the more general context.

The mGMSB model has six parameters [2, 3]: the SUSY breaking scale ($\Lambda$), the mass scale of the SUSY loop messengers ($M_m$), the number of messenger supermultiplets ($N_5$), the ratio of the vacuum expectation values of the two neutral Higgs fields ($\tan \beta$), the sign of the Higgs mass parameter ($\mu$), and the parameter $C_{\text {grav}}$, which affects the gravitino mass. The Spheno3.0 SUSY spectrum generator [20] is used to compute the masses of the $\widetilde{\tau _1}$ as a function of the above six parameters. The SPS7 benchmark scenario [21] is used to determine the parameter space, where $N_5 = 3$, $\tan \beta = 15$, $\mu > 0$, $M_m = 2\Lambda$, and the parameter $C_{\text {grav}} = 4000$ are fixed. Variation of $\Lambda$ then uniquely determines the $\widetilde{\tau _1}$ mass and lifetime, which is of the order of 100 ns. In this study the $\widetilde{\tau _1}$ is considered stable.

**Table 1:** Values of the mGMSB $\Lambda$ parameters in the SPS7 scenario used in this study, the corresponding masses of the $\widetilde{\tau _1}$, $m_{\widetilde{\tau }}$, and the cross-section of the pair production at next-to-leading order. The last two columns give the detector acceptance *A*

$\Lambda$ (TeV)	$m_{\widetilde{\tau }}$ (GeV/c$^2$)	$\sigma$ (fb)		*A* (%)	
		7 TeV	8 TeV	7 TeV	8 TeV
40	124	16.90 $\pm$ 0.79	21.20 $\pm$ 0.91	8.3	9.5
50	154	7.19 $\pm$ 0.38	9.20 $\pm$ 0.46	6.5	7.7
60	185	3.44 $\pm$ 0.20	4.50 $\pm$ 0.24	5.2	6.1
70	216	1.79 $\pm$ 0.11	2.39 $\pm$ 0.14	4.3	5.0
80	247	1.00 $\pm$ 0.07	1.35 $\pm$ 0.08	3.4	4.1
90	278	0.57 $\pm$ 0.04	0.80 $\pm$ 0.05	2.8	3.4
100	309	0.34 $\pm$ 0.02	0.49 $\pm$ 0.03	2.3	2.9

The predictions for $$\widetilde{\tau _1}$$ pair production are based on next-to-leading order (NLO) cross-section calculations by the Prospino2.1 program [22] using the CTEQ6.6M parton distribution function (PDF) set [23]. These predictions at $$\sqrt{s}$$$$=$$ 7 and 8  TeV are presented in Table 1. The relative theoretical uncertainties vary between 5 and 8 %, and are determined following Ref. [24].

Fully simulated signal samples, with masses varying from 124 to 309 GeV/$$c^2$$, have been produced for proton–proton collisions at $$\sqrt{s}$$$$=$$ 7 and 8  TeV. The $$\widetilde{\tau _1}$$ pairs generated by Pythia 6.423 [25], with both $$\widetilde{\tau _1}$$ particles in the fiducial range $$1.8 < \eta < 4.9$$ are passed to Geant4  [26, 27] for detector simulation. The fraction of $$\widetilde{\tau _1}$$ pairs within the fiducial range is defined as the acceptance, *A*. The acceptance factor obtained from Pythia with the MSTW2008 PDF set [28] is also shown in Table 1, with model uncertainties ranging from 5 to 9 % for $$\widetilde{\tau _1}$$ mass from 124 to 309 $${\mathrm {\,GeV\!/}c^2}$$, mainly associated to the choice of PDF.

For larger $\widetilde{\tau _1}$ masses, the Drell–Yan process results in a lower forward boost of the $\widetilde{\tau _1}$ pair, with a subsequent increase in the pair opening angle in the detector frame. The decrease of *A* for an increasing $\widetilde{\tau _1}$ mass is due to a higher probability for one of the particles to escape the LHCb geometrical acceptance.

### Background

The main background is from the Drell–Yan production of muon pairs, $${\mathrm {Z}}/\gamma ^{\star } \rightarrow \mu ^{+} \mu ^{-}$$. Samples of $$Z/\gamma ^{\star } \rightarrow \mu ^{+} \mu ^{-}$$ events have been produced with Pythia and fully simulated with Geant4. The cross-section for this process has been calculated with DYNNLO [29] at next-to-next-to-leading order with the MSTW2008 PDF set. The preselection requirements (see Sect. 4.1) lead to values of the predicted cross-section in LHCb for $$\sqrt{s}$$$$=$$ 7 and 8  TeV of $$1.08 \pm 0.10$$ and $$1.36 \pm 0.12$$ pb, respectively. These values are nearly two orders of magnitude larger than the predicted $$\widetilde{\tau _1}$$ pair cross-section in the most favourable case, corresponding to $$\Lambda =40$$ Te V.

Other background sources include muons produced by top quark pairs, and from $\tau$ pairs. To study the background contributions from these processes, samples of ${\mathrm {Z}}/\gamma ^{\star } \rightarrow \tau ^{+} \tau ^{-}$ and top quark pair decays have been simulated.

## Data selection

The event selection is performed in two steps: a preselection aimed at suppressing the most prominent backgrounds, followed by a multivariate analysis, based on an artificial neural network that is trained using calibrated simulation.

### Preselection

CMSP candidates are identified as high-momentum charged particles with hits in the VELO, all the tracking stations and the four last muon detectors.

Events are selected that contain two or more such particles where one of the particles passes the high-$$p_\mathrm{T}$$ single muon trigger with a threshold of 15 $${\mathrm {\,GeV\!/}c}$$. The trigger efficiency is estimated from simulation to be 92 % for a mass of 124 $${\mathrm {\,GeV\!/}c^2}$$, and 89 % for 309 $${\mathrm {\,GeV\!/}c^2}$$. The two candidates must have opposite charge and each have $$p_\mathrm{T}$$$$>50$$ $${\mathrm {\,GeV\!/}c}$$. To reject background from $$Z/\gamma ^{\star } \rightarrow \mu ^{+} \mu ^{-}$$ the pair must have a dimuon mass larger than 100 $${\mathrm {\,GeV\!/}c^2}$$. A mass-dependent lower threshold on momentum is applied to select particles with $$\beta > 0.8$$.

Several criteria are used to reject muons, electrons and hadrons. Pions and kaons in jets may be identified as muons if they decay in flight or if shower fragments escape from the calorimeters to the muon stations. As hadrons and electrons deposit more energy in the calorimeters than that expected for CMSPs, an efficient rejection of these backgrounds is achieved by requiring the sum of the ECAL and HCAL energies associated with the extrapolation of the charged particle to the calorimeters to be less than 1 % of the momentum of that particle. The background from misidentified muons contributes approximately equally to same- and opposite-charge pairs [30]. No same-charge event is found in the preselected data, showing that this contribution is negligible.

CMSPs, as well as muons from ${\mathrm {Z}}/\gamma ^{\star }$ decays, would be produced at the PV and should have a smaller IP with respect to the PV than muons from heavy quark or tau decays. Requiring an IP of less than 50 $\upmu$m selects efficiently CMSP candidates. After preselection, the contribution from the ${\mathrm {Z}}/\gamma ^{\star } \rightarrow \tau ^{+} \tau ^{-}$ process where both taus decay leptonically to muons is estimated from simulation to contribute less than 0.1 events in total. Pairs of muons produced from top quark decays into *b* quarks and $W^{\pm }$ bosons, with the $W^{\pm }$ bosons decaying leptonically into muons, contribute less than one event, as determined from simulation.

In summary, after preselection the only significant source of background is from $${\mathrm {Z}}/\gamma ^{\star } \rightarrow \mu ^{+} \mu ^{-}$$ decays. The predicted number of dimuon events in the 7  Te V(8  TeV) data set is 249 $$\pm $$ 49 (570 $$\pm $$ 110) which is in good agreement with the 239 (713) observed candidate events. The uncertainties comprise contributions from the preselection cuts (Sect. 6), and the uncertainty on the $${\mathrm {Z}}/\gamma ^{\star } \rightarrow \mu ^{+} \mu ^{-}$$ cross-section (Sect. 3.2). The expected number of events with $$\widetilde{\tau _1}$$ pairs is 2.7 events in the full data set of $$\mathcal {L}$$$$=$$ 3.0 $$bad hbox^{-1}$$, according to the cross-section calculated with Prospino2.1, with SPS7 parameters and a $$\widetilde{\tau _1}$$ mass of 124 $${\mathrm {\,GeV\!/}c^2}$$.

### Selection

An artificial neural network (ANN) is used to distinguish CMSPs from muons by exploiting the difference in interactions that these particles have in matter. To reduce model dependence, the ANN is applied to the individual CMSP candidates, rather than to CMSP-pairs, and a minimum requirement is placed on the product of the two ANN responses. Four variables of the CMSP candidates are used as ANN inputs, computed from the energy deposited in the VELO sensors ($\Delta$E VELO), in the ECAL ($\Delta$E ECAL), in the HCAL ($\Delta$E HCAL), and a likelihood variable associated with the RICH information (DLLx). Model dependence is reduced as much as possible by the absence of kinematical observables in the ANN. The energy loss of a charged particle traversing a VELO sensor follows a Landau distribution. The most probable energy deposition in a sensor is estimated using a truncated mean where only the 60 % lowest depositions are averaged.

**Table 2:** Number of events with both CMSPs candidates satisfying $\mathrm DLLx>-5$, and the final efficiency, $\epsilon$, after the multivariate analysis selection, given for each mass hypothesis

$m_\mathrm{CMSP}$ (${\mathrm {\,GeV\!/}c^2}$)	CMSP-pair candidates		$\epsilon$ (%)	
	7 TeV	8 TeV	7 TeV	8 TeV
124	38	73	49.6 $\pm$ 4.4	45.1 $\pm$ 4.4
154	36	68	48.9 $\pm$ 4.5	44.5 $\pm$ 4.5
185	36	68	46.0 $\pm$ 4.7	41.9 $\pm$ 4.7
216	28	56	42.0 $\pm$ 4.8	38.5 $\pm$ 4.8
247	24	49	37.5 $\pm$ 5.0	35.0 $\pm$ 5.0
278	24	49	32.8 $\pm$ 5.1	31.2 $\pm$ 5.1
309	13	30	28.4 $\pm$ 5.3	27.3 $\pm$ 5.3

Particle identification for a CMSP candidate, using RICH information, is provided by the DLLx variable. A particle identification hypothesis is assigned to a track using a likelihood method. The information from the three radiators is combined and a “delta log-likelihood” (DLL) value computed. The DLL gives, for each track, the change in the overall event log-likelihood when the particle ID hypothesis is changed from $\pi$ to $\mu$, *e*, K, p. The DLLx classification has been added to account for high momentum particles which do not radiate, or have a Cherenkov angle which is too small to fit one of the five particle hypotheses. A positive DLLx indicates a high probability that the candidate has a relatively low velocity. More details are given in Sect. 5.

Events with both candidate CMSPs with $\mathrm DLLx >-5$ are used in the analysis, with no loss of signal, as deduced from simulation. The numbers of selected events with CMSP-pairs are given in Table 2.

**Fig. 2:**
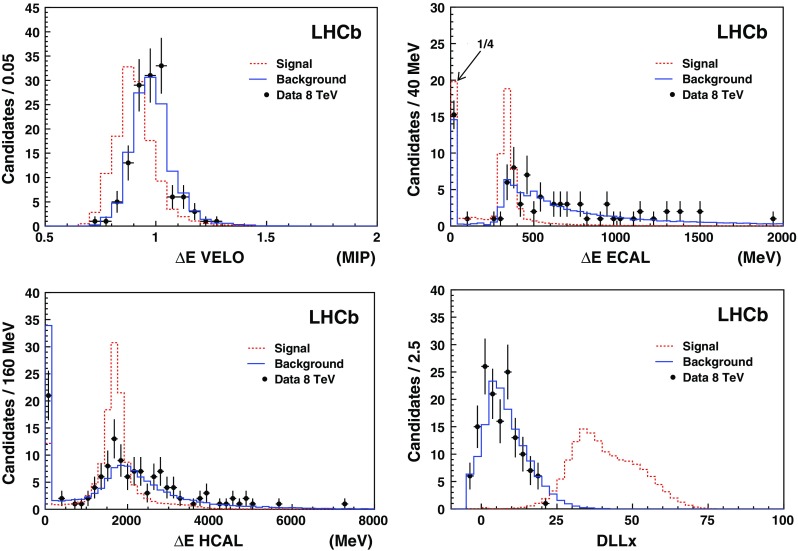
Number of CMSP candidates, as a function of the four variables used as inputs to the ANN. There are two CMSP candidates per event. The *black dots with error bars* show the 2012 data. The *dashed red histogram* is the expected shape for 124 ${\mathrm {\,GeV\!/}c^2}$ CMSPs and the *blue histogram* shows the background from ${\mathrm {Z}}/\gamma ^{\star }$ decays into muons. The energy in the VELO is given in units of minimum ionising particle (MIP) deposition. The first bin of the histogram for $\Delta$E in the ECAL has been multiplied by a factor 0.25

**Fig. 3:**
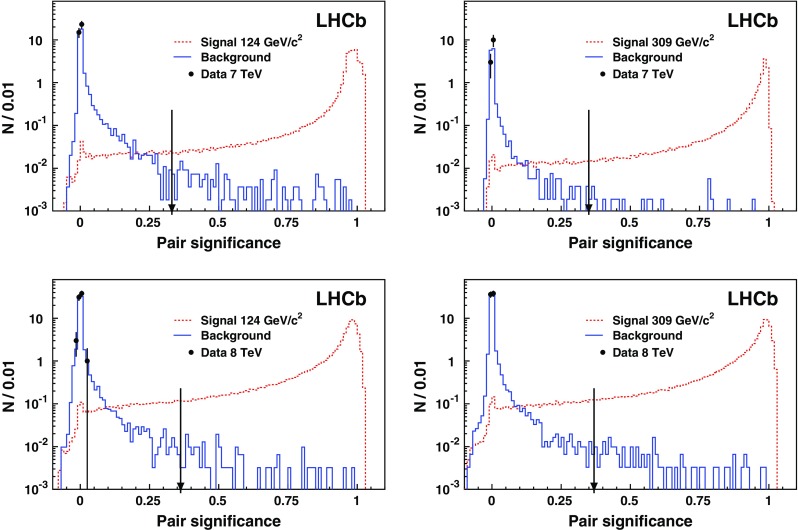
Number of CMSP pairs, *N*, as a function of the pair significance. The *left and right figures* correspond to the CMSP masses of 124 and 309 ${\mathrm {\,GeV\!/}c^2}$, respectively. The *black points* with their statistical uncertainty show the 7 TeV (*top*) and 8 TeV (*bottom*) data sets. The *red dashed histogram* is the expected shape from CMSP pairs and the *blue histogram* the background; both are normalised to the number of events. The *arrows* indicate the chosen selection criteria

Simulated events are used to train the ANN. The first three variables defined above are calibrated using muons from ${\mathrm {Z}}/ \gamma ^{\star } \rightarrow \mu ^{+} \mu ^{-}$ to ensure that simulation agrees with data. A total of 25k $\mathrm {Z}$ events from the 2011 data set and 65k from the 2012 data set are used for the calibration. In the $\mathrm {Z}$ mass region the expected amount of signal is smaller than one event and cannot bias the procedure. The DLLx variable is by far the most discriminating, and its calibration procedure is presented in detail in Sect. 5.

The ANN training is carried out independently for the 7 and 8  Te Vdata sets, and for all the CMSP mass hypotheses considered. Figure 2 shows the distribution of the four ANN input variables for the 8  Te Vdata set, compared to the background and signal predictions; good agreement can be seen between data and simulated background.

The discriminating variable is the product of the ANN outputs obtained for the two CMSP candidates. This “pair significance” is shown in Fig. 3 for the 124 and 309 ${\mathrm {\,GeV\!/}c^2}$ CMSP mass hypotheses for both 7 and 8  Te Vdata sets. The requirement placed on the pair significance is determined by the value needed to achieve a 95 % signal efficiency. After the pair significance selection, the signal efficiency for candidate events in the LHCb acceptance is 50 % for CMSPs with a mass of 124 ${\mathrm {\,GeV\!/}c^2}$, decreasing for increasing mass to a minimum of 27 % at 309 ${\mathrm {\,GeV\!/}c^2}$. The signal efficiency values for CMSPs in the acceptance, after the ANN selection, are given in Table 2. After the full selection is applied, the dimuon background is suppressed by a factor of $10^{-5}$.

## CMSP identification with Cherenkov detectors

The present study uses the Cherenkov radiation produced in the RICH detectors to identify CMSPs. The Cherenkov momentum thresholds for muons, protons, and CMSPs with masses of 124 and 309 ${\mathrm {\,GeV\!/}c^2}$, are given in Table 3 for the three radiators in the LHCb detectors. Only CMSP candidates with momenta above 200 ${\mathrm {\,GeV\!/}c}$ are considered. For this momentum range, particles with masses of the order of ${\mathrm {\,MeV\!/}c^2}$ to ${\mathrm {\,GeV\!/}c^2}$, have Cherenkov angles very close to the saturation value $\arccos (1/(n \beta ))$, where *n* is the refractive index of the medium. The fraction of CMSPs with momentum above 2 ${\mathrm {\,TeV\!/}c}$ is negligible, and the CMSPs are therefore expected not to produce Cherenkov radiation in the gaseous radiators. Around half of the 124 ${\mathrm {\,GeV\!/}c^2}$ CMSPs have a momentum above the Cherenkov threshold for aerogel, and Cherenkov angles smaller than the saturation value. This allows them to be separated from the background. Only a few percent are expected to be in the momentum range 1.4–2.0 ${\mathrm {\,TeV\!/}c}$, corresponding to Cherenkov angles from 0.225 to 0.234 $\mathrm \,rad$. It is possible to distinguish these angles from the saturation value of 0.242 $\mathrm \,rad$ in the aerogel as the angular resolution is about 5.6 $\mathrm \,mrad$.

As previously said, the variable DLLx has been introduced to identify high momentum particles which do not radiate, or have a Cherenkov angle which is too small to fit one of the five particle hypotheses, $\pi$, $\mu$, *e*, K, p. The DLLx value is positive for the momentum distributions of the CMSPs, for all of the masses considered.^1^

**Table 3:** Refractive indices and Cherenkov $\beta$ thresholds for the three radiators. The momentum threshold is given for muons, protons, and 124 and 309 ${\mathrm {\,GeV\!/}c^2}$ CMSPs

			*p* $_\mathrm{thresh}$ (${\mathrm {\,GeV\!/}c}$)			
Radiator	*n*	$\beta _\mathrm{thresh}$	$\mu$	p	CMSP(124)	CMSP(309)
Aerogel	1.03	0.9709	0.428	3.8	502	1252
$\mathrm C_4F_{10}$	1.0014	0.9985	2.00	17.7	2342	5069
$\mathrm CF_4$	1.0005	0.9995	3.34	29.7	3921	9767

Simulated dimuon background events and CMSP signal samples used to train the ANN are first validated with data.

**Fig. 4:**
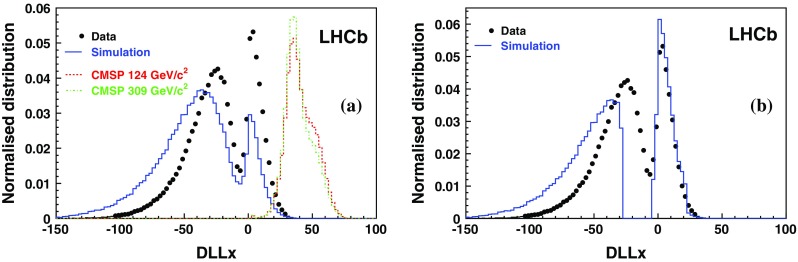
**a** DLLx distributions from muons selected from *Z* decays in 7 TeV data (*black points*) and simulation (*blue histogram*). The expected shape from 124 and 309 ${\mathrm {\,GeV\!/}c^2}$ CMSPs are also shown. **b** The simulated DLLx values are shifted in such a way that data and simulation have the same fraction of entries with $\mathrm {DLLx}>-5$

The study of the background samples is performed on a set of muons above the Cherenkov threshold and selected from $\mathrm {Z}$ decays. Such events have an event topology and kinematics that are very close to those of the dimuon background expected in the CMSP analysis. The DLLx distribution is shown in Fig. 4a for muons from data and simulated $\mathrm {Z}$ decays. For illustration, the expected signal shapes for CMSPs with masses of 124 and 309 ${\mathrm {\,GeV\!/}c^2}$ are superimposed. The small difference is due to a change in the underlying event and some light from the aerogel for the 124 ${\mathrm {\,GeV\!/}c^2}$ case. A clear separation between the signal and background muon DLLx distributions is observed. The difference in the data and simulated muon distributions is mainly due to the lack of precision in the mapping of the photon detection efficiency in the RICH system. In particular, the peak at $\mathrm {DLLx}>-5$ is produced by the decrease of the photon detection efficiency when approaching boundaries in the RICH modules. The simulation only partially reproduces this behaviour and the number of candidates above $\mathrm {DLLx}=-5$ is too low by around a factor of two. To compensate for this, 15 % of simulated muon events with DLLx falling close to zero have been shifted by an*ad hoc* value to obtain the best agreement between data and simulation in the $\mathrm {DLLx}>-5$ region, resulting in the distribution shown in Fig. 4b. It is expected that a correct efficiency map should produce such a shift, moving above zero the slightly negative DLLx values. This set of simulated background events is used to train the ANN. Note that only candidates with $\mathrm {DLLx}>-5$ are used in the ANN. In order to assess the systematic uncertainty associated to this correction method, two other procedures are considered. In the first procedure, the ANN training is performed on the original background simulated data set. In the second, the DLLx values for each muon are randomly chosen following a set of templates inferred from the DLLx data distributions as function of *p* and $\eta$. Despite the fact that this operation is done in bins of *p* and $\eta$, it is obvious that most of the correlation is lost in the randomisation process. The three methods are found to provide the same final discrimination power and their contributions to the systematic uncertainties are small. This is due to the strong separation between signal and background that is provided intrinsically by the DLLx variable.

The validation of the signal sample is more complex due to the absence of a SM process that can be used for calibration. The quality of the simulation was studied using protons from ${\Lambda } \rightarrow \mathrm{p} \pi$ decays with a velocity below the Cherenkov threshold. The differences between the data and simulation for these protons are extrapolated to the CMSP kinematics using a fast simulation method, and the contribution to the systematic uncertainty estimated.

The proton is below the Cherenkov threshold in all of the RICH radiators for *p*$<3.8\,{\mathrm {\,GeV\!/}c}$, and above the threshold for *p*$\gtrsim 30\,{\mathrm {\,GeV\!/}c}$. Pairs of opposite-charge tracks are selected from data and from simulated events passing a minimum bias trigger. The pair must combine to form a particle with a mass compatible with the known mass of the $\Lambda$ baryon, and the reconstructed vertex must be more than 3 $\mathrm \,mm$ from the beam axis.

**Fig. 5:**
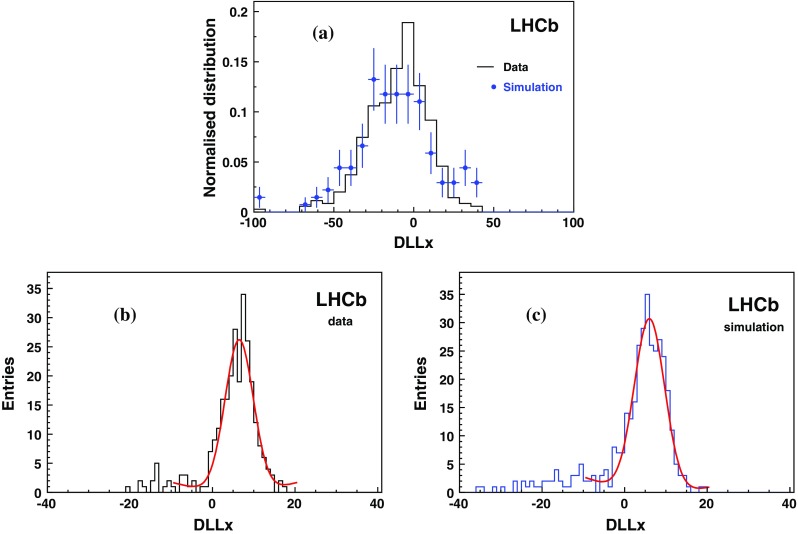
The DLLx variable for: **a** protons selected above the Cherenkov threshold, *p* $$ > 30$$ $${\mathrm {\,GeV\!/}c}$$, in 7 TeV data (*black line*) and simulation (*blue points*), **b** and **c** below-threshold protons, *p* $$ < 3.8{\mathrm {\,GeV\!/}c} $$, from data and simulation, respectively. The results from the fit of a Gaussian function plus polynomial to the below-threshold proton distributions are shown by the *red curves*

Samples of protons below and above Cherenkov threshold are obtained by choosing the momentum regions below 3.8 ${\mathrm {\,GeV\!/}c}$ and above 30 ${\mathrm {\,GeV\!/}c}$, respectively. Figure 5 shows the corresponding DLLx distributions, indicating a reasonable agreement between the DLLx distributions from data collected at 7  TeV and simulation.

A Gaussian function plus a polynomial, to account for the tail at low DLLx, is fitted to the DLLx distributions for below-Cherenkov-threshold particles. The mean and width of the Gaussian functions are $6.5\pm 0.3$ and $3.4\pm 0.3$ for data, and $6.0\pm 0.3$ and $3.6\pm 0.4$ for simulation. The DLLx value in data is $0.5\pm 0.4$ units higher, which may indicate that there is a lower photon detection efficiency in data compared to simulation. A maximal deviation of $\pm$1 DLLx units is considered in the following to assess the systematic effects. The extrapolation from the low momentum proton result to the CMSP regime is made using a fast simulation.

In addition to the Geant4-based full simulation, a fast simulation describing the main features of the RICH measurement process is also used. This allows the impact of varying parameters and the algorithms to be studied in a more efficient way. The fast simulation generates a target particle (a proton from $\Lambda$ decays, a muon or a CMSP) with a momentum distribution representing the phenomenon under study. The underlying event is represented by a number of pions with a momentum distribution obtained from minimum bias events. The simulation of Cherenkov emission in the radiators is then performed for each particle. The number of Cherenkov photons generated by a particle of velocity $\beta$ follows a Poisson distribution of average $\mathrm{N_0} (\beta ^2 n^2 -1)/(\beta ^2 (n^2 -1))$, where $\mathrm N_0$ is the maximal number of photons for a saturated ring and *n* is the refractive index. The ring has an average radius corresponding to the expected Cherenkov angle and a Gaussian profile of width $\sigma _c$ representing the angular resolution of the detector. Finally, random noise is added using the probability for a pixel to fire, $\mathrm prob_{noise}$. The nominal values of the parameters used in the fast simulation are given in Table 4. The event log-likelihood for each target particle hypothesis is

1 $$\begin{aligned} \mathrm LL = -\sum _{\text {pixel}\, i}^{\text {all pixels}}\nu _i + \sum _{\text {pixel}\, i}^{\text {active pixels}} \ln {(e^{\nu _i}-1)} \end{aligned}$$

where $\nu _i$ is the probability to have photons in the pixel *i*, including the random background. Note that the formula is valid for the binary readout implemented in the RICH electronics. The centre of each Cherenkov ring is defined by the true particle direction. The DLL values are subsequently computed.

**Table 4:** Nominal values of the parameters used in the fast simulation

Parameter	Aerogel	$\mathrm C_4F_{10}$	$\mathrm CF_{4}$
*n*	1.03	1.0014	1.0005
$\mathrm N_0$	8	28	24
$\sigma _c$ (mrad)	5.6	1.6	0.7
$\mathrm prob_{noise}$ (%)	3	3	3

**Fig. 6:**
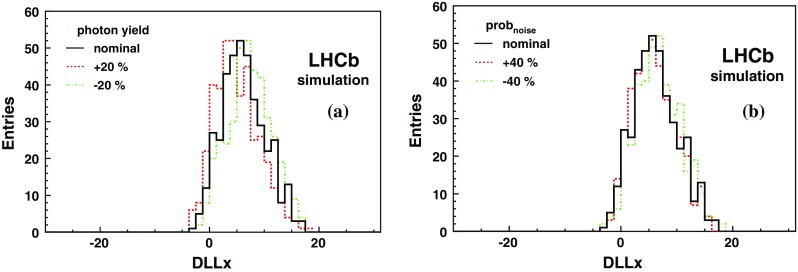
DLLx for protons with $p < 3.8{\mathrm {\,GeV\!/}c}$. In *black* the result with nominal simulation parameters are shown. **a** The *red, dashed, and green, dash-dot*, plots are for a change by $+20$ and *-20* % of the photon yield, **b** is for a change by $+40$ % (*red, dashed*) and $-40$ % (*green, dash-dot*) of the random noise probability

Simulated distributions for protons from $\Lambda$ decays with $p < 3.8{\mathrm {\,GeV\!/}c}$ are shown in Fig. 6. The average DLLx is 6.2, with an RMS of 4.0, for a fast simulation made using the nominal parameters. Figure 6a also shows the distributions after varying the detection efficiency by $\pm$20 %. The corresponding distributions are shifted by $\mp 1.2$ units. The random noise probability was changed by $\pm$40 % from its nominal value which produces negligible variation as seen in Fig. 6b. This study shows that a variation of $\pm$1 DLLx units is obtained by changing the photon detection efficiency by $\mp$15 %. A variation of the same size can be obtained by changing the angular resolution $\sigma _c$ by 50 %.

The DLLx distributions for CMSPs obtained from the fast simulation with nominal parameters are consistent with those obtained from full simulation. Changing the photon detection efficiency by $\pm$15 %, as inferred from the $\Lambda$ study, a variation of $\pm$2 DLLx units is obtained. An identical result can be obtained by changing the angular resolution.

In summary, an uncertainty of two DLLx units is inferred from the comparison of below-threshold protons in data and simulation when using $\Lambda$ decays. The extrapolation to the CMSP regime is obtained by varying the simulation parameters and leads to an uncertainty of four DLLx units on the average DLLx value for such particles.

## Uncertainties and results

After the ANN selection the signal prediction for the chosen model is 2.5, 0.9 and 0.3 events for the 124, 154 and 185 $${\mathrm {\,GeV\!/}c^2}$$$$\widetilde{\tau _1}$$ masses, and below 0.1 for the other mass values. The expected background is negligible, less than 0.02 events for all the mass hypotheses.

A summary of the systematic uncertainties is given in Table 5. The total systematic uncertainties are approximately 5 % for the signal yield and 50 % for the background yield.

**Table 5:** Systematic uncertainties (in %) for the selection of the signal of CMSP pairs and on the background retention. Where relevant, the lower value corresponds to the CMSP mass of 124 ${\mathrm {\,GeV\!/}c^2}$ and the higher one to 309 ${\mathrm {\,GeV\!/}c^2}$

	7 Te V		8 Te V	
	Signal efficiency	Background retention	Signal efficiency	Background retention
Luminosity	1.7	1.7	1.2	1.2
Trigger, reconstruction	2.0	2.0	2.0	2.0
Delayed signals	2.2–3.7	0	2.2–3.7	0
IP calibration	0	0.7	0	0.7
Hadron electron rejection	0	15	0	15
Neural network	2.9	50.0	2.9	50.0
Total system uncertainty	4.5–5.4	52.3	4.3–5.2	52.3

Two methods are used to determine the luminosity: a Van der Meer scan and a beam-gas imaging method [31]. The uncertainties on the integrated luminosities are 1.7 % for the 7  Te Vdata set and 1.2 % for the 8  TeV data set.

The efficiency for triggering, reconstructing and identifying high-$p_\mathrm{T}$ muons has been studied in detail for the LHCb ${\mathrm {Z}}$ and ${\mathrm {W}}$ boson cross-section measurements [32, 33], and the agreement between data and simulation was found to be better than 2 %. This percentage is taken as the corresponding uncertainty for this analysis.

A further efficiency uncertainty is considered due to the delayed signals in the tracking and muon systems. The timing precision affects the amplitude recorded by the front-end electronic boards and the measurement of the drift time in the straw tubes. The effect on the efficiency due to a timing uncertainty of $\pm$1 ns is determined from simulation as a function of the $\beta$ of the particle. Subsequently, a weighted average of the uncertainty is obtained from the $\beta$ distributions for each mass hypothesis, providing values varying from 2.2 to 3.7 % for CMSP masses from 124 to 309 ${\mathrm {\,GeV\!/}c^2}$.

The comparison of the IP distributions in data and simulated ${\mathrm {Z}}/\gamma ^{\star } \rightarrow \mu ^{+} \mu ^{-}$ events indicates a maximal discrepancy of $\pm {5}~\upmu$m. By changing the requirement on the IP parameter by this amount, the corresponding efficiency variation is $\pm$0.7 % for the background and negligible for the signal.

The hadron and electron rejection is affected by the calibration of the calorimeters. From the comparison of ${\mathrm {Z}}/\gamma ^{\star } \rightarrow \mu ^{+} \mu ^{-}$ decays in data and simulation a relative uncertainty of 10 % is inferred. This translates into a 15 % change on the background yield, while the signal is almost unaffected.

The training of the ANN is affected by the uncertainty on the background and signal models. A 2.7 % contribution to the signal efficiency uncertainty is associated with the calibration procedures, determined by the comparison of data and simulation for ${\mathrm {Z}}/\gamma ^{\star } \rightarrow \mu ^{+} \mu ^{-}$ and ${\Lambda } \rightarrow \mathrm {p} \pi$ decays. Error propagation is performed by modifying the ANN training sets, while keeping the test sets and the pair significance selection fixed. Adding the uncertainties in quadrature with the statistical uncertainty of 1 %, gives a total of 2.9 %.

The ANN selection leaves a very small amount of simulated background. The binomial uncertainty on the background retention is large, at approximately 50 %. This value is assigned as the uncertainty on the background selection efficiency.

As already stated, the acceptance A is affected by model uncertainties in the range from 5 to 9 % for $\widetilde{\tau _1}$ mass from 124 to 309 ${\mathrm {\,GeV\!/}c^2}$. In addition, the choice of the PDF affects the efficiency by modifying the momentum of the CMSP. By scanning various PDFs, we have found that this effect is small, not larger that 0.4 %, for all the models.

**Fig. 7:**
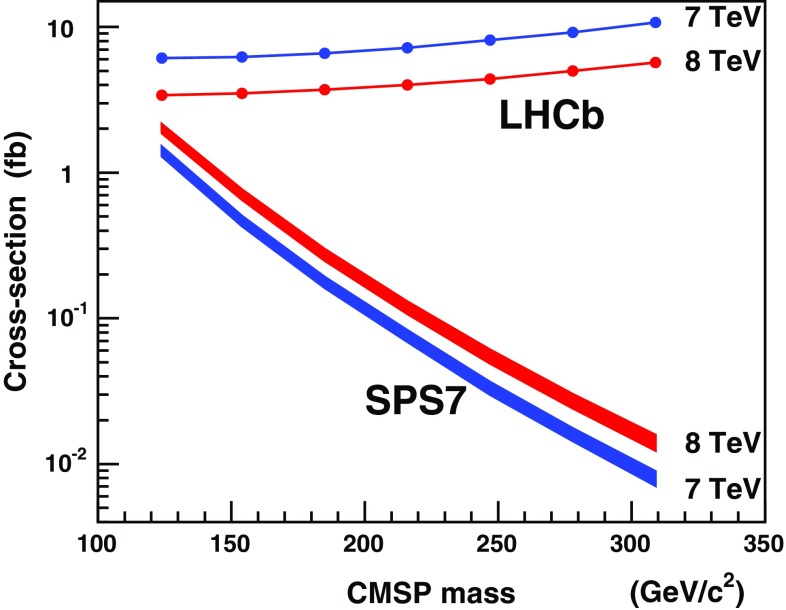
Upper limits at 95 % CL on the cross-sections for the pair production of CMSPs in the LHCb acceptance (*points*) and the corresponding predictions assuming the Drell–Yan production of $\widetilde{\tau _1}$ (*bands* representing $\pm {1} \sigma$ uncertainty) with SPS7 parameters, for proton–proton collisions as a function of the CMSP mass at $\sqrt{s}$ $=$ 7 and 8 TeV

The cross-section upper limits are computed using the Feldman–Cousins method [34] for zero observed candidates, taking into account the expected number of background events and the uncertainties [35]. The predicted amount of background is so small that it has no sizeable effect on the result. The upper limits at a 95 % confidence level (CL) for CMSP pair production in the LHCb geometrical acceptance at $$\sqrt{s}$$$$=$$ 7 and 8  TeV are listed in Table 6 and shown in Fig. 7 together with the theoretical cross-sections calculated for the particular model described in Sect. 3.1.

**Table 6:** Cross-section upper limits at 95 % CL for CMSP pair production in the LHCb acceptance in the 7 and 8 TeV

$m_\mathrm{CMSP}$ (${\mathrm {\,GeV\!/}c^2}$)	Upper limit (fb)	
	7 Te V	8 Te V
124	6.1	3.4
154	6.2	3.5
185	6.6	3.7
216	7.2	4.0
247	8.1	4.4
278	9.2	5.0
309	10.7	5.7

## Conclusions

A search for pairs of long-lived charged particles, with masses in the range 124–309 $${\mathrm {\,GeV\!/}c^2}$$, using $$\widetilde{\tau _1}$$ pairs predicted by the mGMSB model as a benchmark scenario, is performed using data from proton–proton collisions at 7 and 8  Te V, corresponding to an integrated luminosity of 3.0 $$bad hbox^{-1} $$, collected with the LHCb detector in the forward pseudorapidity range $$1.8 <\eta <4.9$$. The candidates are assumed to interact only through the electroweak interaction in the detector. Hence, they behave like heavy muons and their main signature is the absence of a signal in the RICH detectors. The detection efficiency is limited to particles with $$\beta > 0.8$$ due to the acceptance in time after beam crossing. The main background contribution comes from $${\mathrm {Z}}/\gamma ^{\star } \rightarrow \mu ^{+}\mu ^{-}$$ and is reduced to less than $$\sim $$0.02 events. No events have been observed. Upper limits are set on the Drell–Yan CMSP pair production cross-section. For proton–proton collisions at $$\sqrt{s}$$$$=$$ 7  Te V, the 95 % CL upper limits for the production cross-section of a pair of CMSPs in the LHCb acceptance vary from 6.1 fb for a mass of 124 $${\mathrm {\,GeV\!/}c^2}$$ up to 10.7 fb for a mass of 309 $${\mathrm {\,GeV\!/}c^2}$$. At $$\sqrt{s}$$$$=$$ 8  Te V, they vary from 3.4 to 5.7 fb for the same masses.

In LHCb the identification of CMSPs relies on the energy deposited in the subdetectors, the main discrimination power being provided by the RICH system. Together with the forward pseudorapidity coverage, this unique feature allows LHCb to complement the searches undertaken by the central detectors at the Tevatron and LHC.

## Footnotes

1 An anomalous signature in the RICH detectors could also be produced by tracks with wrongly assigned momenta. This can happen if the particle has an absolute electric charge that is different from the proton charge. For instance, a 1 ${\mathrm {\,GeV\!/}c^2}$ proton-like particle is expected to produce Cherenkov light in the two gaseous radiators when the measured momentum is above 30 ${\mathrm {\,GeV\!/}c}$. If the particle has one third of the proton charge, the measured momentum is overestimated by a factor of three and this will lead to an incorrect calculation of the Cherenkov emission.
